# Association Between Socioeconomic Status and Neuropsychiatric Symptoms in the UK Biobank: The Moderating Role of Sociability

**DOI:** 10.1155/da/1293449

**Published:** 2025-05-15

**Authors:** Jiahui Xiao, Bingqing Guo, Yuxin Ma, Ninghao Huang, Tao Huang, Hailun Liang

**Affiliations:** ^1^School of Public Administration and Policy, Renmin University of China, Beijing, China; ^2^School of Public Health, LKS Faculty of Medicine, The University of Hong Kong, Hong Kong SAR, China; ^3^Department of Epidemiology and Biostatistics, School of Public Health, Peking University, Beijing, China; ^4^School of Population and Health, Renmin University of China, Beijing, China

**Keywords:** genetic risk scores, neuropsychiatric symptoms, sociability, socioeconomic status

## Abstract

**Introduction:** Neuropsychiatric symptoms are signs of cognitive decline and associated disorders. The effects of socioeconomic status and social interaction on cognitive decline have already been well documented. Accordingly, the present study aimed to build on the work investigating those factors and cognitive health by examining the relationships between socioeconomic status, sociability, and neuropsychiatric symptoms.

**Methods:** Data from the UK Biobank (*N* = 301,848) were subjected to logistic regressions to examine the relationship between socioeconomic status, sociability, and neuropsychiatric symptoms and sociability to identify any potential moderator in the socioeconomic status-neuropsychiatric symptoms relationship. Specifically, socioeconomic status was defined by the Townsend deprivation index, while sociability was constructed using a cumulative score of four aspects. Meanwhile, neuropsychiatric symptoms were represented by depression, anxiety, and irritability, each of which had a genetic risk score calculated.

**Results:** Individuals who reported lower socioeconomic status also reported more depression and anxiety, while those with higher sociability reported fewer depression and anxiety. Further, it was found that sociability moderated the relationship between socioeconomic status and two symptoms: depression and anxiety. No significant moderating effects were found regarding irritability.

**Conclusion:** The study results indicate the need for interventions aimed at neuropsychiatric symptoms to reduce possible cognitive disorders. They also demonstrate the need to eliminate economic and social disparities and the importance of improving sociability.

## 1. Introduction

The prevalence of cognitive impairment has emerged as a critical public health challenge worldwide. According to the World Health Organization (WHO), over 55 million people are living with dementia globally, with nearly 10 million new cases were diagnosed each year [1]. Neuropsychiatric symptoms, particularly affective and emotional symptoms, including depression, anxiety, and irritability, are not only a core manifestation of dementia and mild cognitive impairment [2–5] but also early markers of cognitive impairment [6]. To better design effective interventions aimed at improving cognitive health, it is imperative to identify socioeconomic status population groups at the highest risk of developing neuropsychiatric symptoms.

A growing body of research from diverse countries and regions has demonstrated that socioeconomic status significantly influences the prevalence of neuropsychiatric symptoms. In these studies, socioeconomic status has been operationalized using various indicators, including educational attainment, income or financial stress, housing conditions, and employment status. Systematic reviews have consistently identified unemployment, lack of housing property, and financial difficulties as global risk factors for depression [7, 8]. For instance, a meta-analysis focusing on perinatal depression in China found that poor educational attainment, low income, inadequate housing conditions, and unemployment were significant risk factors [9]. Low income and low educational attainment were also risk factors of anxiety and depression in the UK general population during the COVID-19 pandemic [10, 11]. Additionally, low job security was also found to be a risk factor of anxiety and depression in United States during and after the COVID-19 pandemic [12].

The strong association between low socioeconomic status and the increased prevalence of neuropsychiatric symptoms underscores significant inequalities in cognitive health. In response, the global research community has intensified efforts to identify coping strategies that may mitigate the onset of neuropsychiatric symptoms and associated cognitive impairment. Among these strategies, sociability has emerged as a promising protective factor against neuropsychiatric symptoms. For instance, a study conducted in Austria during the COVID-19 lockdown demonstrated that sociability significantly reduced the likelihood of mental disorders [13]. Furthermore, a global systematic review identified sociability as a protective factor against depression among postpartum women [14], while another systematic review focusing on Mainland China highlighted its role in mitigating perinatal depression among Chinese women [9]. Besides the direct effects of sociability on neuropsychiatric symptoms, a small number of studies also explored the associations between socioeconomic status, sociability, and neuropsychiatric symptoms. A longitudinal study among Finnish young adults found low level of social support, a relevant concept of sociability, had a greater impact on depression among lower socioeconomic status groups [15]. A longitudinal study among Chinese older adults found that low socioeconomic status were associated with poorer cognitive ability but social support could moderate the relationship between socioeconomic status and cognitive ability [16].

### 1.1. Socioeconomic Status and Neuropsychiatric Symptoms

One widely recognized theoretical explanation deployed in this area is the cognitive reserve theory [17]. Cognitive reserve should be relevant to brain damages. It refers to the brain's ability to optimize or maximize performance through efficient use of brain networks or the recruitment of alternate networks in response to damages [18]. All individuals would experience negative changes or damages with increased age, occurrence of disease, or other damages in life course. These damages would increase individuals' risk of suffering from cognitive impairment, but individuals with greater cognitive reserve could better cope with these risks [19]. The possible mechanisms are as follows: First, according to the *passive model*, facing the same cognitive function decline, individuals with greater cognitive reserve are less likely to reach the threshold of being diagnosed with cognitive impairment. Second, according to the *active model*, individuals with greater cognitive reserve could better use brain networks, recruit alternate networks, and use brain structures or networks not engaged when the brain is not damaged more effectively [17]. Therefore, they could tolerate a larger lesion than others before impairment is apparent and also have greater resilience when damage occurs.

Cognitive reserve theory states that cognitive reserve is accumulated through life course, including people's lifelong choices and lifetime experiences [20]. Several socioeconomic status represent these lifelong choices and experiences. The most well-known factor is education. Education provides individuals with more effective strategies and problem-solving skills, therefore lead to more efficient use of brain networks and alternate networks when facing cognitive difficulties [21]. Based on panel data from the Health and Retirement Study in the United States, a study found higher educational attainment had a lifelong positive effect on cognitive function, with an indirect effect through enhancing cognitive reserve [22]. Among black individuals, education quality moderated the association between disability and cognitive function, showing that high education quality could buffer the cognitive damages due to disability [23]. Occupational attainment plays a similar role with education, individuals with more cognitive demanding occupations are exposure to more cognitive activities, thus, more likely to have greater cognitive reserve. This mechanism has been supported by empirical analyses socioeconomic status from France [24], Australia [25], and The United States [26]. The community or regional environment is also an important component of socioeconomic status. More affluent communities or regions tend to provide more and better community centers as well as more stable neighbors, thus encouraging individuals to participate in more social and physical activities. These activities could further improve an individual's cognitive reserve [27, 28]. Income, one of the most important socioeconomic status factors, is typically associated with higher educational attainment and more cognitive demanding occupational attainment, thus is also linked with greater cognitive reserve [29, 30].

### 1.2. The Role of Sociability

Theoretically, cognitive reserve theory underscores that sociability is another vital factor influencing cognitive health. Sociability is a key aspect of personality, reflecting the tendency and ability of an individual to engage in social interactions, relationships, and social activities [31]. On the one hand, sociability directly serve as a protective factor of cognitive impairment as well as the associated neuropsychiatric symptoms. Better sociability improves brain structure, which results in the more efficient use of brain networks and greater cognitive reserve [20, 32]. It was also significantly associated with better global cognition and various aspects of cognition, such as episodic memory, attention, and processing speed [33]. On the other hand, sociability is potentially a factor that moderates the association between socioeconomic status and cognitive impairment [15, 16, 34]. As mentioned, both high socioeconomic status and sociability could improve individuals' cognitive reserve. Individuals with low socioeconomic status would probably rely more on alternate protective factors, such as sociability and associated social activities, interactions, and relationships. A longitudinal study among Chinese older adults found that compared with individuals with low social interactions, a smaller disparities in cognitive function between low and high socioeconomic status individuals were observed among individuals with high social interactions [16].

**Figure 1:**
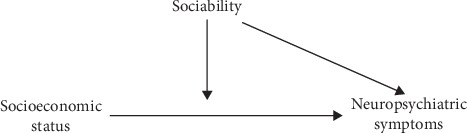
Diagram for the relationship between socioeconomic status, sociability, and neuropsychiatric symptoms.

Regarding the link between the above factors and cognitive health, previous studies have tended to focus on measurable cognitive decline or disease outcomes, such that less attention has been paid to neuropsychiatric symptoms as early marker of cognitive decline and disease progression. Moreover, most studies have focused on only one aspect of sociability, such as social activities, without integrating the proactive feature of sociability [35]. Despite substantial evidence supporting the protective effects of sociability on cognitive impairment, only a limited number of studies have explored the moderating role of sociability in the association between socioeconomic status and neuropsychiatric symptoms [16]. With the above in mind, the present study investigated the relationships between socioeconomic status, sociability, and neuropsychiatric symptoms, as well as testing how sociability moderates the relationship between socioeconomic status and neuropsychiatric symptoms. Exactly as the relationship illustrated in Figure 1, we hypothesized that socioeconomic status and sociability could directly impact the incidence of neuropsychiatric symptoms based on cognitive reserve, and sociability would serve as a moderator between socioeconomic status and neuropsychiatric symptoms.

## 2. Methods

### 2.1. Participants

The study data were derived from the UK Biobank, a sizable biomedical database and research resource. We used cross-sectional data from the baseline survey of the UK Biobank. It contains detailed genetic and health information from 500,000 UK participants. We included all participants of the UK Biobank who were sampled between 2006 and 2010. The response rate was 5.5%. The age range is 37–73 years old. The UK Biobank invited around 9.2 million participants registered with the National Health Service (NHS) to attend one of 22 assessment centers across England, Scotland, and Wales to complete touch-screen questionnaires, undergo physical measurement, and provide biological samples.

### 2.2. Measures

#### 2.2.1. Socioeconomic Status

The Townsend deprivation index was designed by Townsend et al. [36]. It is a simple census-based index of material deprivation calculated using the combination of four census variables. Since its first use, this measure has been widely used in the field. The Townsend index is an area-based measure that can be constructed for any geographical area for which census data are available. The Townsend deprivation index was computed using data from the previous national census, which included information on household overcrowding, automobile ownership, home ownership, and unemployment as a percentage of the population. We divided the participants into two groups: participants with scores above the median were grouped into high subgroups, while participants with scores below the median were grouped into low subgroups.

We included income as a socioeconomic status variable, as suggested by previous studies [29, 30]. UK Biobank covered the average total income before tax received by the respondent's household. It was collected in five categories: less than 18,000 £, 18,000 £ to 30,999 £, 31,000 £ to 51,999 £, 52,000 £ to 100,000 £, and more than 100,000 £. We kept these five categories and included it as an ordinal categorical variable in our analyses.

We included educational attainment as an ordinal categorical variable in our analysis status based on previous analyses [22, 23]. We categorized each individual's educational attainment into four levels: higher education, secondary education, work-related practical qualifications, and none of the above.

#### 2.2.2. Sociability

Based on each participant's overall score on four questions drawn from the UK Biobank that reflect several complimentary components of sociability [37], we defined sociability as follows [20, 32]: (1) “How often do you visit friends or family or have them visit you?”; (2) “Which of the following (leisure or social activities) do you attend once a week or more often?”. The options include sports club or gym, pub or social club, religious group, adult education class, other group activity, and none of the above; (3) “Do you worry too long after an embarrassing experience?”; and (4) “Do you often feel lonely?” are questions regarding the frequency of social gatherings. The scale for sociability scores is 0–4. It should be noted that participants were excluded if they answered “No friends/family outside household” (as these individuals could not answer the question about the frequency of visits), “Do not know,” or “prefer not to answer” to any of the questions, or if they had somatic problems that could impair their sociability (narcolepsy, stroke, severe tinnitus, deafness, or brain-related cancers). As noted above, the participants were categorized into two subgroups—high-score and low-score groups—based on the median of their sociability scores (2.80). For details, please see Table S1.

#### 2.2.3. Neuropsychiatric Symptoms

We first measured the phenotype of neuropsychiatric symptoms. Neuropsychiatric symptoms include a variety of symptoms; however, because of the limited number of cases of other symptoms in hospital diagnostic records, three symptoms were selected as dependent variables: anxiety, depression, and irritability. The 9^th^ revision of International Classification of Diseases (ICD-9) codes 311 and 300, respectively, as well as ICD-10 codes F32–F33 and F40–F41, were used to characterize depression and anxiety in hospital records. Meanwhile, irritability was defined by ICD-10 codes R45.4.

Neuropsychiatric symptoms are partially determined by genetics [38]. Thus, it is also important to account for genetic factors of neuropsychiatric symptoms while assessing the association between socioeconomic status and neuropsychiatric symptoms. Recent development in genome-wide association studies provides a useful tool, genetic risk score, to measure the genetics factors of health outcomes in a more comprehensive way [39]. We calculated the genetic risk score of each neuropsychiatric symptoms: anxiety, depression, and irritability, following the standard calculation process [39]. Previous genome-wide association studies have identified that 9 single nucleotide polymorphisms are strongly associated with anxiety, 37 single nucleotide polymorphisms are strongly associated with depression, and 22 single nucleotide polymorphisms are strong associated with irritability [40, 41]. Detailed information about single nucleotide polymorphisms included in this study can be found in the Table S2. We extracted data on these single nucleotide polymorphisms of UK Biobank samples from Integrative Epidemiology Unit open genome-wide association studies project. Each nucleotide polymorphism was recoded as 0, 1, or 2 and treated as a separate risk factor. Next, the linkage disequilibrium test, Hardy–Weinberg equilibrium test, and stand flip were carried out in our data and confirmed the validity of these single nucleotide polymorphisms in our samples. Subsequently, the genetic risk score of anxiety was calculated by adding together unweighted values of 9 associated single nucleotide polymorphisms. The same calculation was done for 37 single nucleotide polymorphisms associated with depression as well as the 22 single nucleotide polymorphisms associated with irritability. Lastly, three categorical variables were created for anxiety, depression, and irritability genetic risk score, respective. Each variable was categorized into three groups: low genetic risk group (quintile 1), middle genetic risk group (quintiles 2–4), and high genetic risk group (quintile 5).

#### 2.2.4. Covariates

We included several demographical characteristics, including age, gender, and ethinicity. The participants were categorized into three age groups: under 55, between 55 and 65, and above 65, based on the date of their birth. Gender is also included as a covariate. The UK Biobank surveyed respondents from multiple ethnicities. We included ethnicity as a categorical covariate, grouping participants into white, south Asian, Black, Chinese, mixed, and others.

We included several variables to reflect participant's health status since it is associated with neuropsychiatric symptoms [42]. To assess if a participant was obese, their BMI was taken into consideration. Participants were classified as being normal weighted (18.5≤BMI <25), underweight (BMI <18.5), overweight (25≤BMI <30), and obese (BMI≥30). We also included several cognitive disorders since they are strongly associated with neuropsychiatric symptoms: Alzheimer's disease, dementia, mild cognitive impairment, and Parkinson's disease [43].

Also, family history was included as a covariate. If any of the participants' parents, siblings, or other family members had ever had depression, that family member's history of neuropsychiatric symptoms was deemed to exist.

In addition, sleep quality score and lifestyle score were calculated and categorized. The total of five dichotomous variables—sleep duration, early chronotype, insomnia, snoring, and daytime dozing—constituted the sleep score. If a participant satisfied one or two criteria, their sleep state was graded as “unfavorable,” “medium” if they met two or three, and “favorable” if they met four or more. The lifestyle score was determined by taking into account things including obesity, alcohol consumption, smoking cessation, and diet. The overall scores ranged from 0 to 4. A higher score represents a better lifestyle. This variable was included into the analysis as a categorical variable. It was calculated by summing four elements up. Each element was recorded as 0 or 1. Participants with a BMI between 18.5 and 24.9 and a waist circumference of less than 88 cm for women and less than 102 cm for men were classified as healthy, while the rest of the participants increased their lifestyle score by one point. Participates who drank more than 14 g of alcohol per day for women and more than 28 g per day for men were codes as 1 on alcohol consumption. Furthermore, individuals who have never smoked increased their lifestyle score by one point. A healthy diet explicitly considers an individual's intake of bread, cereal, fruit, vegetables, fish, and meat.

### 2.3. Statistical Analysis

In the first step, the prevalence of anxiety, depression, and irritability was examined, and the characteristics of the participants were summarized using descriptive methods based on those subgroups with neuropsychiatric symptoms and those without neuropsychiatric symptoms. In the second step, the respective roles of socioeconomic status and sociability were analyzed using four separate logistic models for each of the dependent variables. In each set of analyses, Model 1 included the socioeconomic status, sociability, and control variables, while Models 2, 3, and 4 further added interaction term between each socioeconomic status variable and sociability based on Model 1, the purpose of which was to examine the moderating role of sociability. It should be noted that the demographic characteristics, health status, family history, lifestyle, sleep scores, and the genetic risk score of each symptom were controlled in all models.

For severe class imbalance for irritability (58 positive cases vs., 301,790 negative cases), we applied undersampling to improve the balance (1:10 ratio) and retained all positive samples for analysis. And firth penalized logistic regression was employed to mitigate separation issues caused by rare events. Each model for irritability presents the median of the OR values from 50 times of under-sampling Firth logistic regression, the proportion of significance (*p*-value < 0.05) and the proportion of positive effects.

We did subgroup analyses for participants aged 55–65 years as well as ≥65 years to provide implications for aging populations.

All analyses were carried out using Stata SE 16.0.

## 3. Result

### 3.1. Sample Characteristics

A total of 301,848 participants were finally included in this study after those with missing data were removed. The prevalence of three kinds of neuropsychiatric symptoms and the characteristics of the participants are detailed in Table 1.

In total, 16,963 individuals (5.62%) were exposed to depression, 29,029 individuals (9.62%) were exposed to anxiety, and 83,928 individuals (27.8%) reported feelings of irritability. White people (ethnicity group) have the highest prevalence of neuropsychiatric symptoms, while the mixed group has the lowest prevalence of neuropsychiatric symptoms. Compared to participants without neuropsychiatric symptoms, those with any kind of neuropsychiatric symptoms were more likely to be in the age group <55 years old, have a family history of depression, lead a less healthy lifestyle, experience poorer sleep quality, and have a higher corresponding GRS for each symptom. These features showed a high degree of commonality across the individuals sampled, regardless of whether they had experienced depression, anxiety, or irritability. A total of 276,641 individuals (91.65%) are white people, 10,365 individuals are south Asian (3.43%), 10,264 individuals are Black (3.40%), 1317 individuals are Chinese (0.44%), 784 individuals are mixed (0.26%), and 2477 individuals are others (0.82%).

### 3.2. The Association of Socioeconomic Status and Sociability With Neuropsychiatric Symptoms

The hierarchical analysis results are set out in Tables 2–4.  Table 2 shows the logistic regression models for depression. Model Ⅰ included all covariates. Incorporating socioeconomic status, Model 2 identified a significant association between deprivation and depression, indicating that those with higher level of deprivation at baseline were significantly more likely to experience depression, adjusting for all covariates (OR = 1.326, *p* < 0.001). Similarly, compared with the poorest group, richer participants were significantly less likely to experience depression (OR = 0.220 to 0.568, *p*  < 0.01). It also shows that participants with higher educational attainment as the baseline were less likely to suffer from depression (OR = 0.742 to 0.864, *p*  < 0.001). Model 3 further included sociability and indicated that a those with high sociability score at baseline were significantly less likely to experience depression, adjusting for socioeconomic status and all covariates (OR = 0.344, *p* < 0.001).  Table 4 shows the results of logistic regression models for anxiety. Similar to the results for depression, higher level of deprivation was associated with a higher risk of anxiety (OR = 1.223, *p*  < 0.001) according to Model 2. Similarly, participants with higher income (OR = 0.329 to 0.655, *p*  < 0.01) and educational attainment (OR = 0.800 to 0.892, *p*  < 0.01) at the baseline were less likely to suffer from anxiety. As shown in Model 3, higher sociability score was associated with a lower risk of anxiety (OR = 0.398, *p*  < 0.001).

In Table 3, the association between socioeconomic status as well as sociability and irritability is insignificant. Take Model 1 for example, only 50% of analyses reported positive effects, which was not enough to have a concise finding.

### 3.3. The Moderating Role of Sociability

Tables 2–4 detailed the results on interaction terms between each socioeconomic status and sociability regarding depression, anxiety, and irritability. As shown in Table 2 Model 2, the results demonstrated that the interactive effect of deprivation and sociability was significantly positive for depression (OR = 0.913, *p*=0.014).  Table 2 Model 3 shows the positive interactive effect of income and sociability (OR = 1.110 to 1.292, *p*  < 0.05), suggesting that the sociability weakened the association between lower income and higher risks of depression. A shown in Model 4 of Table 2, the interaction term between sociability and the highest educational attainment group also shows that sociability weakened the association between low educational attainment and higher risks of depression (OR = 1.145, *p*  < 0.05).  Table 4 shows similar moderating effects of sociability could be found for anxiety. Table 3 shows that regarding irritability, no significant moderating effects were found.

### 3.4. Subgroup Analysis

Tables S3–S8 shows the results for subgroup analysis regarding depression, anxiety, and irritability, respective. Tables S3–S8 shows the main findings hold for two subgroups as well.

## 4. Discussion

To the best of our knowledge, this is the first study to examine the relationship between socioeconomic status and neuropsychiatric symptoms, with investigating the role of sociability. This study also has unique contributions in controlling the genetic risk score of neuropsychiatric symptoms. The overarching aim of the present study was to investigate the relationships between socioeconomic status, sociability, and neuropsychiatric symptoms. Additionally, it tested the moderating role of sociability between socioeconomic status and neuropsychiatric symptoms. From the analysis detailed above, the results of the descriptive statistics show the baseline characteristics of the population with neuropsychiatric symptoms and to some extent provide a reference for the prevention of neuropsychiatric symptoms and the identification of the population. More to the point, it was found that those individuals who reported lower socioeconomic status reported more depression and anxiety, while those with higher sociability reported fewer depression and anxiety. Further, it was determined that sociability moderated the relationship between socioeconomic status and depression as well as anxiety. These results were consistent with those put forward in the existing research on cognitive health.

First, the direct association between socioeconomic status and neuropsychiatric symptoms was investigated, and the results of which were in line with those from the previous research on cognitive health. Although past studies used different measurements for cognitive status (i.e., the Cog-State brief battery [44], the Montreal Cognitive Assessment [45], and the Mini-Mental State Examination [46]), the findings were nonetheless consistant, which also demonstrates the reliability of neuropsychiatric symptoms in predicting cognitive decline. Moreover, as the Townsend index is an area-based measure of socioeconomic status, the overall result can be explained by the socio-ecological theory [47]. To be precise, the availability of basic neighborhood infrastructure and physical and social resources (e.g., recreational facilities, community centers, and libraries) promotes cognitively beneficial activities, such as exercise and social integration [27, 48]. Notably, this finding is consistent with cognitive reserve theory.

In addition, the present study found that sociability was a protective factor for reported neuropsychiatric symptoms. The definition of sociability used here was based on the UKBB data and covered a number of aspects of social interaction, including loneliness, social relationships, social embarrassment, and social activities. Prior studies have also provided evidence on how social interaction impacts cognitive health in relation to these various aspects. Both transient and persistent loneliness are significant predictors of cognitive decline, though persistent loneliness shows a stronger association with poor cognitive function [49]. Individuals who experience feelings of loneliness may not be as engaged in cognitively stimulating environments, which is detrimental to brain health [50]. Meanwhile, social embarrassment usually implies social misbehavior [51] and is characterized by social and communicative deficits, restricted interests, and repetitive behaviors [52]. It has also been shown that social networks composed of a diverse range of social relationships are likely to be the most beneficial to cognitive health [53, 54], on the basis that people with a range of social ties or those who are frequently in contact with friends perform better in cognition assessment than those who only socialize with their family [53, 55]. A possible explanation is that friends can bring them more mental stimulation and novel experiences than families. However, there are also results from cohort study showing that the development of cognitive impairment is not related to the presence or absence of social support from family or friends, but only to feelings of loneliness [56]. Similarly, the effect of social activities on cognitive function also differs depending on the activity in question, which is based on the amount of cognitive, physical, and social effort individuals need for engagement [57]. Although the pathways through which these aspects impact cognitive status may differ, they nevertheless exert an influence on each other. For example, embarrassment-processing deficits can impair social relationships [58], and participation in social activities can reduce loneliness [59]. Furthermore, there is research suggesting that they each have a distinct strength of effect on cognitive status [60]. Future studies could look more closely at these different aspects of sociability to examine whether they have the same effect on neuropsychiatric symptoms as has been observed in relation to recognized cognitive impairment.

Supporting our hypothesis, we found that sociability has a significant protective effect on the association between socioeconomic status and neuropsychiatric symptoms. Many studies have confirmed the role of social interaction in moderating socioeconomic differences in health, particularly cognitive or psychological health [15, 61]. Moreover, the observed moderating effect of sociability was slightly more significant among those with low socioeconomic status compared to their high socioeconomic status peers. Provided quantitative research demonstrates that the deprivation gradients in lack of social support are considerably smaller than gradients for household income [62]. Individuals with low socioeconomic status tend to live in poorer quality house with fewer local amenities and worrying environment leading to concerns on their own safety, which are potential barriers for them to set social relationships. Consequently, those people with low socioeconomic status and high sociability can better conquer the barriers actively than those with low socioeconomic status and low sociability, resulting in the significant moderating effect among low socioeconomic status participants. Nevertheless, there is no similar barriers among people with high socioeconomic status.

Our findings show that low socioeconomic status is associated with more neuropsychiatric symptoms among older adults. These findings suggest the potential inequalities in cognitive health in old ages. To improve health equalities, we suggest governments to pay special attention on older adults whose live in poor neighborhoods, who have low income, and who have lower educational attainment. Specifically, to increase older adults' income, we would recommend local governments to boost the uptake of some income support welfare schemes for older adults in the UK. For example, for Pension Credit, an income support scheme in the Great Britain, the uptake rate was below 66% for the past 10 years, with up to 850,000 people over State Pension age still estimated to be missing out in 2019–2020 [63]. Governments could consider several community-based support projects to encourage eligible older adults claim through Pension Credit or other income support schemes. Considering the role of sociability, governments could also establish more social activity centers in poor neighborhoods to encourage more social interactions in these areas.

Based on the findings, a number of practical implications can be articulated. As differences in socioeconomic status have a significant impact on cognitive functioning, efforts should be focused on ameliorating social inequities. Policymakers should take into account disparities in socioeconomic status, whether at the individual level (income or housing) or at the regional level (infrastructure and overall social environment). Taking into account the living environment of the poor, the government, in addition to providing infrastructures, should also improve facilities that provide recreation and socialization to help enhance their cognitive reserves. One study found that for people at high risk of cognitive disorders, merely providing health care might not have a significant impact on disease outcomes, and that daily living support and housing support were more essential for them [64]. In addition, community-based emotional comfort and entertainment services have a significant positive influence on cognitive function of the people in the neighborhood [65]. For those who are already showing signs of cognitive decline, such nonpharmacological interventions can be given in the community by public health professionals or community volunteers, to help those who cannot afford hospitalization.

It should be noted that, as neuropsychiatric symptoms are preexisting manifestations of cognitive disorders, it is too late to intervene by the time the disease is diagnosed. Accordingly, more attention should be paid to the primary prevention of cognitive decline. In practice, this will involve monitoring changes in the sociability of individuals. Interestingly, the elderly are not prominently represented among all individuals with neuropsychiatric symptoms reported compared with diagnosed cognitive disorders. Studies have shown that most neuropsychiatric symptoms tend to appear later during adulthood [66]. Because people begin to experience a couple of functional impairments like dysfunctional social relations, high-risk behaviors, poverty, and difficulties with academic or else occupations during this period comorbid with neuropsychiatric symptoms [67]. Besides, existing research has also found that genetic scores for neuropsychiatric symptoms can affect the onset of symptoms; hence, it makes sense to focus on sociability across all ages. Changes in sociability include changes in social cognition and changes in social behavior. Practitioners must also be mindful that these kinds of dysfunction that signal cognitive decline can be sometimes mistakenly diagnosed as mania or hypomania [68]. As these symptoms are characterized by the co-occurrence of cognitive, emotional, and behavioral abnormalities, family members and friends should be alert to the unusual social behavior of those around them.

### 4.1. Strengths and Limitations

The strength of this study is that it investigated specific symptoms and constructed GRS for depression, anxiety and irritability, which aimed to emphasize the importance of neuropsychiatric symptoms as signs of cognitive decline and disease progression. Additionally, a sociability score was constructed by drawing on different aspects to explore the relationship between socioeconomic status and neuropsychiatric symptoms, which broadened the scope set by past research on social interaction and cognitive health.

An apparent limitation is that the study only included three symptoms as outcome variables. This is a problem as neuropsychiatric symptoms can be manifested as agitation, delusions, hallucinations, depression, euphoria, aberrant motor behavior, apathy, irritability, disinhibition, and anxiety [69]. However, due to data limitations, we were only able to select three of these symptoms as representative. Second, we only tested one moderator. The relationship between socioeconomic status and neuropsychiatric symptoms is complex, and there are many possible pathways. As such, crucial moderators may differ between age groups, for example, after the age of 75 years, while many other factors may moderate or influence the risk of cognitive decline, including frailty, losing the partner, and family-based care. With this in mind, other mechanisms related to socioeconomic status and neuropsychiatric symptoms warrant further investigation. Third, the cohort that completed the mental health questionnaire recorded higher levels of educational attainment and socioeconomic status than the overall UK Biobank cohort and the UK population, which may have influenced the measurement of the relationship between socioeconomic status and neuropsychiatric symptoms. Fourth, the low respondent rate of the UK Biobank (5.5%) might cause selection bias. If people who did not respond had worse cognitive health or lower socioeconomic status, the low respondent rate would bias the relationships among socioeconomics, neuropsychiatric symptoms, and sociability. Fifth, we used Townsend deprivation index as one of the socioeconomic variables, which was measured based on earlier census data and was area based. Therefore, there is potential bias in misclassification or changes over time. However, the findings on Townsend deprivation index were consistent with the findings on other individual level two socioeconomic variables, namely income and educational attainment, which suggesting the validity of the Townsend deprivation index. Sixth, we measured sociability using four items, which was not a well-known scale. Unfortunately, because of the data availability, we were unable to use a better tool in this study. However, these items have been used in previous studies, showing its reliability to some extent [38]. We recommend future studies develop a more comprehensive scale to measure sociability as well as to test its validity and reliability. Seventh, prior research indicates that sociability serves as a protective factor against neuropsychiatric symptoms, and socioeconomic status may attenuate these benefits [70, 71]. Due to space constraints, this study could not examine sociability's effects or SES moderation. We recommend future studies empirically test this framework using UK Biobank or comparable international datasets. Finally, the UK Biobank is a cross-sectional data; therefore, we were unable to do any casual inference. We suggest readers interpret our results carefully.

## 5. Conclusions

This study explored the influence of socioeconomic status and sociability on neuropsychiatric symptoms and examined the moderating effect of sociability on the association between socioeconomic status and neuropsychiatric symptoms. The results indicate a need for interventions directly targeting neuropsychiatric symptoms to reduce possible cognitive disorders. Moreover, the findings highlight the challenges in narrowing economic and social disparities. To respond to these challenges, policy tools are needed to improve the physical environment in less developed areas and develop approaches to support deprived individuals. Finally, it is necessary to pay attention to social cognition and behavior, both of which may imply neuropsychiatric symptoms and improve sociability to increase stress resilience and contribute to cognitive health.

## Figures and Tables

**Table 1:** Descriptive statistics of the study population.

Characteristic	All participants (*n* = 301,848), *N* (%)	Depression		Anxiety		Irritability	
		Yes (*n* = 16,963), *N* (%)	No (*n* = 284,885), *N* (%)	Yes (*n* = 29,029), *N* (%)	No (*n* = 272,819), *N* (%)	Yes (*n* = 58), *N* (%)	No (*n* = 301,790), *N* (%)
Age							
<55	135,177 (44.78)	9655 (56.92)	125,522 (44.06)	16,415 (56.55)	118,762 (43.53)	17 (29.31)	135,160 (44.79)
55–65	115,661 (38.32)	5489 (32.36)	110,172 (38.67)	9394 (32.36)	106,267 (38.95)	15 (25.86)	115,646 (38.32)
≥65	51,010 (16.90)	1819 (10.72)	49,191 (17.27)	3220 (11.09)	47,790 (17.52)	26 (44.83)	50,984 (16.89)
Gender							
Female	159,614 (52.88)	9710 (57.24)	149,904 (52.62)	17,369 (59.83)	142,245 (52.14)	12 (20.69)	159,602 (52.89)
Male	142,234 (47.12)	7253 (42.76)	134,981 (47.38)	11,660 (40.17)	130,574 (47.86)	46 (79.31)	142,188 (47.11)
Household income							
<18,000	58,978 (19.54)	6070 (35.78)	52,908 (18.57)	8737 (30.10)	50,241 (18.42)	21 (36.21)	58,957 (19.54)
18,000–30,999	74,983 (24.84)	4226 (24.91)	70,757 (24.84)	7153 (24.64)	67,830 (24.86)	18 (31.03)	74,965 (24.84)
31,000–51,999	81,436 (26.98)	3772 (22.24)	77,664 (27.26)	7092 (24.43)	74,344 (27.25)	8 (13.79)	81,428 (26.98)
52,000–10,000	67,738 (22.44)	2431 (14.33)	65,307 (22.92)	5050 (17.40)	62,688 (22.98)	8 (13.79)	67,730 (22.44)
>10,000	18,713 (6.20)	464 (2.74)	18,249 (6.41)	997 (3.43)	17,716 (6.49)	(5.17) 3	18,710 (6.2)
Education							
None of the above	40,135 (13.33)	3303 (19.55)	36,832 (12.96)	35,144 (12.91)	4991 (17.26)	13 (22.41)	40,122 (13.33)
Work-related practical qualifications	19,552 (6.49)	1273 (7.53)	18,279 (6.43)	17,516 (6.44)	2036 (7.04)	6 (10.34)	19,546 (6.49)
Secondary education	115,981 (38.52)	6719 (39.76)	109,262 (38.45)	104,211 (38.29)	11,770 (40.7)	25 (43.1)	115,956 (38.52)
Higher education	125,425 (41.66)	5603 (33.16)	119,822 (42.16)	115,300 (42.36)	10,125 (35.01)	14 (24.14)	125,411 (41.66)
Ethnicity							
White	276,641 (91.65)	14,803 (87.27)	261,838 (91.91)	250,680 (91.89)	25,961 (89.43)	57 (98.28)	276,584 (91.65)
South Asian	10,365 (3.43)	839 (4.95)	9526 (3.34)	9149 (3.35)	1216 (4.19)	1 (1.72)	10,364 (3.43)
Black	10,264 (3.4)	768 (4.53)	9496 (3.33)	9113 (3.34)	1151 (3.97)	0 (0)	10,264 (3.4)
Chinese	1317 (0.44)	165 (0.97)	1152 (0.4)	1110 (0.41)	207 (0.71)	0 (0)	1317 (0.44)
Mixed	784 (0.26)	70 (0.41)	714 (0.25)	700 (0.26)	84 (0.29)	0 (0)	784 (0.26)
Others	2477 (0.82)	318 (1.87)	2159 (0.76)	2067 (0.76)	410 (1.41)	0 (0)	2477 (0.82)
Family history of depression							
No	262,043 (86.81)	13,040 (76.87)	249,003 (87.4)	22,917 (78.95)	239,126 (87.65)	44 (75.86)	261,999 (86.82)
Yes	39,805 (13.19)	3923 (23.13)	35,882 (12.6)	6112 (21.05)	33,693 (12.35)	14 (24.14)	39,791 (13.18)
AD							
No	301,443 (99.87)	16,926 (99.78)	284,517 (99.87)	272,469 (99.87)	28,974 (99.81)	50 (86.21)	301,393 (99.87)
Yes	405 (0.13)	37 (0.22)	368 (0.13)	350 (0.13)	55 (0.19)	8 (13.79)	397 (0.13)
Dementia							
No	300,482 (99.55)	16,790 (98.98)	283,692 (99.58)	271,688 (99.59)	28794 (99.19)	46 (79.31)	300,436 (99.55)
Yes	1366 (0.45)	173 (1.02)	1193 (0.42)	1131 (0.41)	235 (0.81)	12 (20.69)	1354 (0.45)
MCI							
No	301,699 (99.95)	16,935 (99.83)	284,764 (99.96)	272,702 (99.96)	28,997 (99.89)	57 (98.28)	301,642 (99.95)
Yes	149 (0.05)	28 (0.17)	121 (0.04)	117 (0.04)	32 (0.11)	1 (1.72)	148 (0.05)
PD							
No	300,845 (99.67)	16,881 (99.52)	283,964 (99.68)	271,984 (99.69)	28,861 (99.42)	54 (93.1)	300,791 (99.67)
Yes	1003 (0.33)	82 (0.48)	921 (0.32)	835 (0.31)	168 (0.58)	4 (6.9)	999 (0.33)
BMI							
Normal weighted	85,880 (28.45)	4084 (24.08)	81,796 (28.71)	78,827 (28.89)	7053 (24.3)	11 (18.97)	85,869 (28.45)
Under weighted	1455 (0.48)	117 (0.69)	1338 (0.47)	1231 (0.45)	224 (0.77)	1 (1.72)	1454 (0.48)
Over weighted	134,948 (44.71)	6573 (38.75)	128,375 (45.06)	123,624 (45.31)	11,324 (39.01)	26 (44.83)	134,922 (44.71)
Obese	79,565 (26.36)	6189 (36.49)	73,376 (25.76)	69,137 (25.34)	10,428 (35.92)	20 (34.48)	79,545 (26.36)
Sleep score							
Unfavorable	12,472 (4.13)	1831 (10.79)	10,641 (3.74)	3150 (10.85)	9322 (3.42)	1 (1.72)	12,471 (4.13)
Medium	173,091 (57.34)	11,367 (67.01)	161,724 (56.77)	19,813 (68.25)	153,278 (56.18)	43 (74.14)	173,048 (57.34)
Favorable	116,285 (38.52)	3765 (22.2)	112,520 (39.50)	6066 (20.90)	110,219 (40.40)	14 (74.14)	116,271 (38.53)
Lifestyle score							
0–1	44,043 (14.59)	4157 (24.51)	39,886 (14.00)	7088 (24.42)	36,955 (13.55)	12 (20.69)	44,031 (14.59)
2	103,116 (34.16)	6214 (36.63)	96,902 (34.01)	10,773 (37.11)	92,343 (33.85)	27 (46.55)	103,089 (34.16)
3	106,305 (35.22)	4860 (28.65)	101,445 (35.61)	8238 (28.38)	98,067 (35.95)	15 (25.86)	106,290 (35.22)
≥4	48,384 (16.03)	1732 (10.21)	46,652 (16.38)	2930 (10.09)	45,454 (16.66)	4 (6.9)	48,380 (16.03)
Sociability score							
Low	147,091 (48.73)	12,784 (75.36)	134,307 (47.14)	20,815 (71.70)	126,276 (46.29)	35 (60.34)	147,056 (48.73)
High	154,757 (51.27)	4179 (24.64)	150,578 (52.86)	8214 (28.30)	146,543 (53.71)	23 (39.66)	154,734 (51.27)
Deprivation index							
Low	188,061 (62.30)	7973 (47.00)	180,088 (63.21)	14,910 (51.36)	173,151 (63.47)	37 (63.79)	188,024 (62.3)
High	113,787 (37.70)	8990 (53.00)	104,797 (36.79)	14,119 (48.64)	99,668 (36.53)	21 (36.21)	113,766 (37.7)
GRS of depression							
Low	100,654 (33.35)	5401 (31.84)	95253 (33.44)	—	—	—	—
Medium	100,812 (33.40)	5606 (33.05)	95206 (33.42)	—	—	—	—
High	100,382 (33.26)	5956 (35.11)	94426 (33.15)	—	—	—	—
GRS of anxiety							
Low	102,585 (33.99)	—	—	9662 (33.28)	92,923 (34.06)	—	—
Medium	103,191 (34.19)	—	—	9953 (34.29)	93,238 (34.18)	—	—
High	96,072 (31.83)	—	—	9414 (32.43)	86,658 (31.76)	—	—
GRS of irritability							
Low	115,856 (38.38)	—	—	—	—	27 (46.55)	115,829 (38.38)
Medium	85,932 (28.47)	—	—	—	—	14 (24.14)	85,918 (28.47)
High	100,060 (33.15)	—	—	—	—	17 (29.31)	100,043 (33.15)

**Table 2:** Logistic regression models for depression.

Variable	Depression							
	Model 1		Model 2		Model 3		Model 4	
	OR	S.E.	OR	S.E.	OR	S.E.	OR	S.E.
Sociability#education								
1#1	—	—	—	—	—	—	1.056	0.084
1#2	—	—	—	—	—	—	1.009	0.053
1#3	—	—	—	—	—	—	1.145	0.061
Sociability#householdincome								
1#1	—	—	—	—	1.110*⁣*^*∗*^	0.054	—	—
1#2	—	—	—	—	1.162*⁣*^*∗∗*^	0.059	—	—
1#3	—	—	—	—	1.262*⁣*^*∗∗∗*^	0.072	—	—
1#4	—	—	—	—	1.292*⁣*^*∗∗*^	0.142	—	—
Sociability#deprivation	—	—	0.913*⁣*^*∗*^	0.034	—	—	—	—
Sociability score (low)								
High	0.344*⁣*^*∗∗∗*^	0.006	0.360*⁣*^*∗∗∗*^	0.009	0.310*⁣*^*∗∗∗*^	0.010	0.326*⁣*^*∗∗∗*^	0.014
Deprivation index (low)								
High	1.307*⁣*^*∗∗∗*^	0.023	1.339*⁣*^*∗∗∗*^	0.027	1.306*⁣*^*∗∗∗*^	0.023	1.306*⁣*^*∗∗∗*^	0.023
Household income (<18,000)								
18,000–30,999	0.576*⁣*^*∗∗∗*^	0.013	0.576*⁣*^*∗∗∗*^	0.013	0.561*⁣*^*∗∗∗*^	0.015	0.576*⁣*^*∗∗∗*^	0.013
31,000–51,999	0.428*⁣*^*∗∗∗*^	0.010	0.428*⁣*^*∗∗∗*^	0.010	0.411*⁣*^*∗∗∗*^	0.011	0.428*⁣*^*∗∗∗*^	0.010
52,000–10,000	0.319*⁣*^*∗∗∗*^	0.009	0.319*⁣*^*∗∗∗*^	0.009	0.300*⁣*^*∗∗∗*^	0.010	0.319*⁣*^*∗∗∗*^	0.009
>10,000	0.226*⁣*^*∗∗∗*^	0.012	0.227*⁣*^*∗∗∗*^	0.012	0.211*⁣*^*∗∗∗*^	0.013	0.227*⁣*^*∗∗∗*^	0.012
Education (None of the above)								
Work-related practical qualifications	0.883*⁣*^*∗∗∗*^	0.032	0.883*⁣*^*∗∗∗*^	0.032	0.883*⁣*^*∗∗∗*^	0.032	0.869*⁣*^*∗∗∗*^	0.037
Secondary education	0.772*⁣*^*∗∗∗*^	0.019	0.772*⁣*^*∗∗∗*^	0.019	0.772*⁣*^*∗∗∗*^	0.019	0.770*⁣*^*∗∗∗*^	0.022
Higher education	0.749*⁣*^*∗∗∗*^	0.020	0.749*⁣*^*∗∗∗*^	0.020	0.749*⁣*^*∗∗∗*^	0.020	0.722*⁣*^*∗∗∗*^	0.022
Age (<55)								
55–65	0.557*⁣*^*∗∗∗*^	0.011	0.556*⁣*^*∗∗∗*^	0.011	0.557*⁣*^*∗∗∗*^	0.011	0.556*⁣*^*∗∗∗*^	0.011
≥65	0.363*⁣*^*∗∗∗*^	0.011	0.362*⁣*^*∗∗∗*^	0.010	0.364*⁣*^*∗∗∗*^	0.011	0.363*⁣*^*∗∗∗*^	0.011
Gender (Female)								
Male	0.911*⁣*^*∗∗∗*^	0.015	0.911*⁣*^*∗∗∗*^	0.015	0.911*⁣*^*∗∗∗*^	0.015	0.913*⁣*^*∗∗∗*^	0.016
Ethnicity (White)								
South Asian	1.268*⁣*^*∗∗∗*^	0.050	1.268*⁣*^*∗∗∗*^	0.050	1.267*⁣*^*∗∗∗*^	0.050	1.268*⁣*^*∗∗∗*^	0.050
Black	1.331*⁣*^*∗∗∗*^	0.054	1.330*⁣*^*∗∗∗*^	0.054	1.331*⁣*^*∗∗∗*^	0.054	1.332*⁣*^*∗∗∗*^	0.054
Chinese	1.881*⁣*^*∗∗∗*^	0.168	1.883*⁣*^*∗∗∗*^	0.168	1.880*⁣*^*∗∗∗*^	0.168	1.882*⁣*^*∗∗∗*^	0.168
Mixed	1.453*⁣*^*∗∗*^	0.193	1.453*⁣*^*∗∗∗*^	0.193	1.455*⁣*^*∗∗*^	0.193	1.454*⁣*^*∗∗*^	0.193
Others	1.907*⁣*^*∗∗∗*^	0.124	1.908*⁣*^*∗∗∗*^	0.124	1.908*⁣*^*∗∗∗*^	0.124	1.910*⁣*^*∗∗∗*^	0.124
Family history of depression (No)								
Yes	1.812*⁣*^*∗∗∗*^	0.036	1.812*⁣*^*∗∗∗*^	0.036	1.812*⁣*^*∗∗∗*^	0.036	1.812*⁣*^*∗∗∗*^	0.036
AD (No)								
Yes	0.855	0.180	0.853	0.179	0.854	0.180	0.854	0.180
Dementia (No)								
Yes	2.260*⁣*^*∗∗∗*^	0.235	2.262*⁣*^*∗∗∗*^	0.235	2.259*⁣*^*∗∗∗*^	0.235	2.261*⁣*^*∗∗∗*^	0.235
MCI (No)								
Yes	2.791*⁣*^*∗∗∗*^	0.643	2.794*⁣*^*∗∗∗*^	0.644	2.801*⁣*^*∗∗∗*^	0.646	2.802*⁣*^*∗∗∗*^	0.646
PD (No)								
Yes	1.388*⁣*^*∗∗*^	0.175	1.389*⁣*^*∗∗*^	0.175	1.388*⁣*^*∗∗*^	0.175	1.388*⁣*^*∗∗*^	0.175
BMI (normal weighted)								
Under weighted	1.133	0.116	1.134	0.116	1.132	0.116	1.134	0.116
Over weighted	0.911*⁣*^*∗∗∗*^	0.022	0.911*⁣*^*∗∗∗*^	0.022	0.910*⁣*^*∗∗∗*^	0.022	0.910*⁣*^*∗∗∗*^	0.022
Obese	1.197*⁣*^*∗∗∗*^	0.030	1.197*⁣*^*∗∗∗*^	0.030	1.197*⁣*^*∗∗∗*^	0.030	1.196*⁣*^*∗∗∗*^	0.030
Sleep score (unfavorable)								
Medium	0.526*⁣*^*∗∗∗*^	0.015	0.526*⁣*^*∗∗∗*^	0.015	0.526*⁣*^*∗∗∗*^	0.015	0.526*⁣*^*∗∗∗*^	0.015
Favorable	0.299*⁣*^*∗∗∗*^	0.010	0.299*⁣*^*∗∗∗*^	0.010	0.299*⁣*^*∗∗∗*^	0.010	0.299*⁣*^*∗∗∗*^	0.010
Lifestyle score (0–1)								
2.000	0.779*⁣*^*∗∗∗*^	0.017	0.779*⁣*^*∗∗∗*^	0.017	0.779*⁣*^*∗∗∗*^	0.017	0.779*⁣*^*∗∗∗*^	0.017
3.000	0.685*⁣*^*∗∗∗*^	0.016	0.685*⁣*^*∗∗∗*^	0.016	0.686*⁣*^*∗∗∗*^	0.016	0.686*⁣*^*∗∗∗*^	0.016
≥4	0.591*⁣*^*∗∗∗*^	0.021	0.591*⁣*^*∗∗∗*^	0.021	0.591*⁣*^*∗∗∗*^	0.021	0.591*⁣*^*∗∗∗*^	0.021
GRS of depression (low)								
Medium	1.028	0.021	1.028	0.021	1.028	0.021	1.028	0.021
High	1.069*⁣*^*∗∗∗*^	0.022	1.068*⁣*^*∗∗∗*^	0.022	1.069*⁣*^*∗∗∗*^	0.022	1.068*⁣*^*∗∗*^	0.022
Constant	0.649	0.031	0.641	0.031	0.667	0.032	0.659	0.032

*⁣* ^*∗*^ represents *p* < 0.05.

*⁣* ^*∗∗*^ represents *p* < 0.01.

*⁣* ^*∗∗∗*^ represents *p* < 0.001.

**Table 3:** Logistic regression models for anxiety.

Variable	Anxiety							
	Model 1		Model 2		Model 3		Model 4	
	OR	S.E.	OR	S.E.	OR	S.E.	OR	S.E.
Sociability#education								
1#1	—	—	—	—	—	—	1.002	0.063
1#2	—	—	—	—	—	—	1.046	0.043
1#3	—	—	—	—	—	—	1.070	0.044
Sociability#householdincome								
1#1	—	—	—	—	1.077	0.041	—	—
1#2	—	—	—	—	1.168*⁣*^*∗∗∗*^	0.045	—	—
1#3	—	—	—	—	1.148*⁣*^*∗∗∗*^	0.049	—	—
1#4	—	—	—	—	1.167*⁣*^*∗*^	0.089	—	—
Sociability#deprivation	—	—	0.931*⁣*^*∗∗*^	0.026	—	—	—	—
Sociability score (Low)								
High	0.398*⁣*^*∗∗∗*^	0.006	0.411*⁣*^*∗∗∗*^	0.008	0.364*⁣*^*∗∗∗*^	0.010	0.382*⁣*^*∗∗∗*^	0.013
Deprivation index (Low)								
High	1.208*⁣*^*∗∗∗*^	0.016	1.235*⁣*^*∗∗∗*^	0.020	1.208*⁣*^*∗∗∗*^	0.016	1.208*⁣*^*∗∗∗*^	0.016
Household income (<18,000)								
18,000–30,999	0.664*⁣*^*∗∗∗*^	0.012	0.664*⁣*^*∗∗∗*^	0.012	0.649*⁣*^*∗∗∗*^	0.014	0.664*⁣*^*∗∗∗*^	0.012
31,000–51,999	0.553*⁣*^*∗∗∗*^	0.011	0.554*⁣*^*∗∗∗*^	0.011	0.527*⁣*^*∗∗∗*^	0.012	0.553*⁣*^*∗∗∗*^	0.011
52,000–10,000	0.459*⁣*^*∗∗∗*^	0.010	0.459*⁣*^*∗∗∗*^	0.010	0.440*⁣*^*∗∗∗*^	0.011	0.459*⁣*^*∗∗∗*^	0.010
>10,000	0.337*⁣*^*∗∗∗*^	0.013	0.337*⁣*^*∗∗∗*^	0.013	0.321*⁣*^*∗∗∗*^	0.014	0.337*⁣*^*∗∗∗*^	0.013
Education (None of the above)								
Work-related practical qualifications	0.909*⁣*^*∗∗∗*^	0.027	0.909*⁣*^*∗∗∗*^	0.027	0.909*⁣*^*∗∗∗*^	0.027	0.909*⁣*^*∗∗∗*^	0.033
Secondary education	0.834*⁣*^*∗∗∗*^	0.017	0.834*⁣*^*∗∗∗*^	0.017	0.834*⁣*^*∗∗∗*^	0.017	0.822*⁣*^*∗∗∗*^	0.020
Higher education	0.807*⁣*^*∗∗∗*^	0.017	0.807*⁣*^*∗∗∗*^	0.017	0.807*⁣*^*∗∗∗*^	0.017	0.790*⁣*^*∗∗∗*^	0.020
Age (<55)								
55–65	0.570*⁣*^*∗∗∗*^	0.009	0.570*⁣*^*∗∗∗*^	0.009	0.570*⁣*^*∗∗∗*^	0.009	0.570*⁣*^*∗∗∗*^	0.009
≥65	0.407*⁣*^*∗∗∗*^	0.009	0.406*⁣*^*∗∗∗*^	0.009	0.408*⁣*^*∗∗∗*^	0.009	0.407*⁣*^*∗∗∗*^	0.009
Gender (Female)								
Male	0.764*⁣*^*∗∗∗*^	0.010	0.763*⁣*^*∗∗∗*^	0.010	0.763*⁣*^*∗∗∗*^	0.010	0.764*⁣*^*∗∗∗*^	0.010
Ethnicity (White)								
South Asian	1.075*⁣*^*∗*^	0.036	1.074*⁣*^*∗*^	0.036	1.074*⁣*^*∗*^	0.036	1.074*⁣*^*∗*^	0.036
Black	1.126*⁣*^*∗∗∗*^	0.038	1.126*⁣*^*∗∗∗*^	0.038	1.126*⁣*^*∗∗∗*^	0.038	1.126*⁣*^*∗∗∗*^	0.038
Chinese	1.380*⁣*^*∗∗∗*^	0.112	1.381*⁣*^*∗∗∗*^	0.112	1.379*⁣*^*∗∗∗*^	0.112	1.380*⁣*^*∗∗∗*^	0.112
Mixed	0.986	0.119	0.986	0.119	0.987	0.119	0.987	0.119
Others	1.475*⁣*^*∗∗∗*^	0.086	1.475*⁣*^*∗∗∗*^	0.086	1.474*⁣*^*∗∗∗*^	0.086	1.476*⁣*^*∗∗∗*^	0.086
Family history of depression (No)								
Yes	1.638*⁣*^*∗∗∗*^	0.027	1.638*⁣*^*∗∗∗*^	0.027	1.638*⁣*^*∗∗∗*^	0.027	1.638*⁣*^*∗∗∗*^	0.027
AD (No)								
Yes	1.004	0.181	1.003	0.180	1.005	0.181	1.005	0.181
Dementia (No)								
Yes	1.809*⁣*^*∗∗∗*^	0.168	1.810*⁣*^*∗∗∗*^	0.168	1.807*⁣*^*∗∗∗*^	0.168	1.808*⁣*^*∗∗∗*^	0.168
MCI (No)								
Yes	1.943*⁣*^*∗∗*^	0.424	1.943*⁣*^*∗∗*^	0.424	1.947*⁣*^*∗∗*^	0.425	1.947*⁣*^*∗∗*^	0.425
PD (No)								
Yes	2.104*⁣*^*∗∗∗*^	0.196	2.104*⁣*^*∗∗∗*^	0.196	2.105*⁣*^*∗∗∗*^	0.196	2.104*⁣*^*∗∗∗*^	0.196
BMI (normal weighted)								
Under weighted	1.334*⁣*^*∗∗∗*^	0.104	1.334*⁣*^*∗∗∗*^	0.105	1.333*⁣*^*∗∗∗*^	0.104	1.334*⁣*^*∗∗∗*^	0.104
Over weighted	0.866*⁣*^*∗∗∗*^	0.016	0.866*⁣*^*∗∗∗*^	0.016	0.866*⁣*^*∗∗∗*^	0.016	0.866*⁣*^*∗∗∗*^	0.016
Obese	1.154*⁣*^*∗∗∗*^	0.023	1.154*⁣*^*∗∗∗*^	0.023	1.154*⁣*^*∗∗∗*^	0.023	1.154*⁣*^*∗∗∗*^	0.023
Sleep score (unfavorable)								
Medium	0.458*⁣*^*∗∗∗*^	0.011	0.458*⁣*^*∗∗∗*^	0.011	0.458*⁣*^*∗∗∗*^	0.011	0.458*⁣*^*∗∗∗*^	0.011
Favorable	0.222*⁣*^*∗∗∗*^	0.006	0.222*⁣*^*∗∗∗*^	0.006	0.222*⁣*^*∗∗∗*^	0.006	0.222*⁣*^*∗∗∗*^	0.006
Lifestyle score (0–1)								
2	0.741*⁣*^*∗∗∗*^	0.013	0.741*⁣*^*∗∗∗*^	0.013	0.741*⁣*^*∗∗∗*^	0.013	0.741*⁣*^*∗∗∗*^	0.013
3	0.606*⁣*^*∗∗∗*^	0.012	0.606*⁣*^*∗∗∗*^	0.012	0.606*⁣*^*∗∗∗*^	0.012	0.606*⁣*^*∗∗∗*^	0.012
≥4	0.493*⁣*^*∗∗∗*^	0.014	0.493*⁣*^*∗∗∗*^	0.014	0.493*⁣*^*∗∗∗*^	0.014	0.493*⁣*^*∗∗∗*^	0.014
GRS of anxiety (low)								
Medium	1.020	0.016	1.020	0.016	1.020	0.016	1.020	0.016
High	1.029	0.016	1.029	0.016	1.029	0.016	1.029	0.016
Constant	1.406	0.054	1.392	0.054	1.446	0.057	1.426	0.057

*⁣* ^*∗*^ represents *p* < 0.05.

*⁣* ^*∗∗*^ represents *p* < 0.01.

*⁣* ^*∗∗∗*^ represents *p* < 0.001.

**Table 4:** Firth logistic regression models for irritability.

Variable	Irritability											
	Model 1			Model 2			Model 3			Model 4		
	Median (OR)	Significant proportion	Positive effect proportion	Median (OR)	Significant proportion	Positive effect proportion	Median (OR)	Significant proportion	Positive effect proportion	Median (OR)	Significant proportion	Positive effect proportion
Sociability#education												
1#1	—	—	—	—	—	—	—	—	—	0.0736	6%	100%
1#2	—	—	—	—	—	—	—	—	—	0.350	14%	100%
1#3	—	—	—	—	—	—	—	—	—	0.436	2%	98%
Sociability#householdincome												
1#1	—	—	—	—	—	—	0.548	0	92%	—	—	—
1#2	—	—	—	—	—	—	1.412	0	80%	—	—	—
1#3	—	—	—	—	—	—	0.916	0	56%	—	—	—
1#4	—	—	—	—	—	—	3.934	2%	100%	—	—	—
Sociability#deprivation	—	—	—	1.727	0	94%	—	—	—	—	—	—
Sociability score (Low)												
High	0.517	50%	100%	0.434	56%	100%	0.519	0	98%	1.269	0	78%
Deprivation index (low)												
High	0.707	0	98%	0.558	6%	100%	0.980	2%	96%	0.678	2%	96%
Household income (<18,000)												
18,000–30,999	0.997	0	52%	0.917	0	62%	1.114	0	78%	0.916	0	64%
31,000–51,999	0.695	0	94%	0.700	0	96%	0.616	0	94%	0.710	0	96%
52,000–10,000	0.781	0	88%	0.740	0	90%	0.842	0	76%	0.751	0	82%
>10,000	1.370	0	84%	1.333	0	82%	0.767	0	78%	1.354	0	86%
Education (none of the above)												
Work-related practical qualifications	0.684	0	84%	0.649	2%	92%	0.659	2%	90%	1.683	0	90%
Secondary education	0.869	0	72%	0.791	0	82%	0.767	0	80%	1.315	0	78%
Higher education	0.488	16%	100%	0.446	22%	100%	0.440	26	100%	0.641	0	92%
Age (<55)												
55–65	0.710	0	96%	0.699	0	98%	0.707	0	96%	0.685	0	98%
≥65	2.261	44%	100%	2.341	54%	100%	2.411	60%	100%	2.224	44%	100%
Gender (Female)												
Male	3.408	100%	100%	3.320	100%	100%	3.364	100%	100%	3.449	100%	100%
Ethnicity (White)												
South Asian	0.488	0	92%	0.411	0	90%	0.428	0	94%	0.423	0	94%
Black	0.960	0	56%	1.029	0	54%	0.908	0	58%	1.030	0	54%
Chinese	3.101	2%	96%	1.777	2%	80%	2.530	2%	85%	2.210	4%	85%
Mixed	5.535	0	90%	3.255	4%	94%	5.266	2%	95%	3.733	3%	95%
Others	1.086	0	54%	1.396	0	66%	1.381	0	62%	1.339	0	62%
Family history of depression (No)												
Yes	3.019	100%	100%	2.908	98%	100%	2.823	94%	100%	3.017	100%	100%
AD (No)												
Yes	4.804	2%	88%	5.822	0	92%	7.223	2%	96%	6.491	0	94%
Dementia (No)												
Yes	17.595	98%	100%	10.090	100%	100%	19.195	100%	100%	25.200	100%	100%
MCI (No)												
Yes	19.335	10%	98%	23.000	14%	100%	22.100	12%	100%	13.680	0	100%
PD (No)												
Yes	5.575	20%	100%	6.701	18%	100%	6.892	26%	100%	5.429	0	100%
BMI (normal weighted)												
Under weighted	5.546	2%	100%	7.865	0	100%	8.812	4%	100%	9.189	4%	100%
Over weighted	1.130	0	64%	1.155	0	74%	1.184	0	86%	1.218	0	80%
Obese	1.591	0	100%	1.634	0	98%	1.654	0	98%	1.687	0	96%
Sleep score (unfavorable)												
Medium	2.241	0	100%	1.745	2%	94%	1.802	2%	98%	1.754	0	96%
Favorable	1.638	0	86%	1.424	2%	82%	1.464	2%	82%	1.476	0	82%
82%Lifestyle score (0–1)												
2	1.127	0	76%	1.070	0	70%	1.110	0	64%	1.123	0	78%
3	0.499	8%	100%	0.494	16%	100%	0.484	16%	100%	0.498	16%	100%
≥4	0.408	0	100%	0.406	0	100%	0.415	0	100%	0.412	0	100%
GRS of irritability (low)												
Medium	1.175	2%	86%	1.152	0	72%	1.178	0	78%	1.126	0	76%
High	0.951	0	60%	0.927	0	64%	0.953	0	58%	0.937	0	64%
Constant	655	—	—	654	—	—	654	—	—	654	—	—

## Data Availability

The data that support the findings of this study are openly available in UK Biobank at https://biobank.ndph.ox.ac.uk/showcase/search.cgi, reference number 44430.

## Supporting Information

Supporting Information Table S1. Phenotype and score of sociability in UK Biobank. Table S2. Characteristics of genetic variants associated with depression, anxiety and irritability in UK Biobank. Table S3. Results of logistic regression analysis for depression in the middle-aged subgroup (45−59 years) (*n* = 146,579). Table S4. Results of logistic regression analysis for anxiety in the middle-aged subgroup (45−59 years) (*n* = 146,579). Table S5. Results of logistic regression analysis for irritability in the middle-aged subgroup (45−59 years) (*n* = 146,579). Table S6. Results of logistic regression analysis for depression in the middle-aged subgroup (≥60 years) (*n* = 121,564). Table S7. Results of logistic regression analysis for anxiety in the middle-aged subgroup (≥60 years) (*n* = 121,564). Table S8. Results of logistic regression analysis for irritability in the middle-aged subgroup (≥60 years) (*n* = 121,564).
