# Supplementary material for: Association Between Socioeconomic Status and Neuropsychiatric Symptoms in the UK Biobank: The Moderating Role of Sociability
Source: Depress Anxiety. 2025 May 15;2025:1293449. doi: 10.1155/da/1293449 (PMC12097854; doi:10.1155/da/1293449)
Supplement: Supporting Information — Table S1. Phenotype and score of sociability in UK Biobank. Table S2. Characteristics of genetic variants associated with depression, anxiety and irritability in UK Biobank. Table S3. Results of logistic regression analysis for depression in the middle-aged subgroup (45−59 years) (n = 146,579). Table S4. Results of logistic regression analysis for anxiety in the middle-aged subgroup (45−59 years) (n = 146,579). Table S5. Results of logistic regression analysis for irritability in the middle-aged subgroup (45−59 years) (n = 146,579). Table S6. Results of logistic regression analysis for depression in the middle-aged subgroup (≥60 years) (n = 121,564). Table S7. Results of logistic regression analysis for anxiety in the middle-aged subgroup (≥60 years) (n = 121,564). Table S8. Results of logistic regression analysis for irritability in the middle-aged subgroup (≥60 years) (n = 121,564). [file 1293449.f1.pdf]

Table S1 Phenotype and score of sociability in UK Biobank

QUESTIONS and SCORING

1. Frequency of friend / family visits (Data-Field 1031)

How often do you visit friends or family or have them visit you? (Hint: If this varies, please give an average of how often you visit or have had visits in the last year. Include meeting with friends or family in environments outside of the home such as in the park, at a sports field, at a restaurant or pub.)

ANSWER	SCORE
1. Almost daily	1
2. 2 - 4 times a week	0.8
3. About once a week	0.6
4. About once a month	0.4
5. Once every few months	0.2
6. Never or almost never	0
7. No friends/family outside household	- exclude
8. Do not know	- exclude
9. Prefer not to answer	- exclude

2. Leisure / social activities (Data-Field 6160)

Which of the following do you attend once a week or more often? (You can select more than one) (Hint: If this varies, please think about activities in the last year.)

ANSWER	SCORE
1. Sports club or gym	At least one of these answers: 1
2. Pub or social club	
3. Religious group	
4. Adult education class	
5. Other group activity	
6. None of the above	0
7. Prefer not to answer	- exclude

3. Worry after social embarrassment (Data-Field 2000)

Do you worry too long after an embarrassing experience?

ANSWER	SCORE
1. Yes	0
2. No	1
3. Do not know	- exclude
4. Prefer not to answer	- exclude

4. Loneliness (Data-Field 2020)

Do you often feel lonely?

ANSWER	SCORE
1. Yes	0
2. No	1
3. Do not know	- exclude
4. Prefer not to answer	- exclude

Table S2 Characteristics of genetic variants associated with depression, anxiety and irritability in UK Biobank

Trait	SNP	CHR	Position	P value	Allele 1	Allele 2	OR	SE	Frequency
Depression	rs1432639	1	72813218	4.6×10^{-15}	A	C	1.04	0.005	0.63
	rs12129573	1	73768366	4.0×10^{-12}	A	C	1.04	0.005	0.37
	rs2389016	1	80799329	1.0×10^{-8}	T	C	1.03	0.0053	0.28
	rs4261101	1	90796053	1.0×10^{-8}	A	G	0.97	0.005	0.37
	rs9427672	1	197754741	3.1×10^{-8}	A	G	0.97	0.0058	0.24
	rs11682175	2	57987593	4.7×10^{-9}	T	C	0.97	0.0048	0.52
	rs1226412	2	157111313	2.4×10^{-8}	T	C	1.03	0.0059	0.79
	rs7430565	3	158107180	2.9×10^{-9}	A	G	0.97	0.0048	0.58
	rs34215985	4	42047778	3.1×10^{-9}	C	G	0.96	0.0063	0.24
	rs11135349	5	164523472	1.1×10^{-9}	A	C	0.97	0.0048	0.48
	rs4869056	5	166992078	6.8×10^{-9}	A	G	0.97	0.005	0.63
	rs9402472	6	99566521	2.8×10^{-8}	A	G	1.03	0.0059	0.24
	rs10950398	7	12264871	2.6×10^{-8}	A	G	1.03	0.0049	0.41
	rs12666117	7	109105611	1.4×10^{-8}	A	G	1.03	0.0048	0.47
	rs1354115	9	2983774	2.4×10^{-8}	A	C	1.03	0.0049	0.62
	rs10959913	9	11544964	5.1×10^{-9}	T	G	1.03	0.0057	0.76
	rs7856424	9	119733595	8.5×10^{-9}	T	C	0.97	0.0053	0.29
	rs7029033	9	126682068	2.7×10^{-8}	T	C	1.05	0.0093	0.07
	rs61867293	10	106563924	7.0×10^{-10}	T	C	0.96	0.0061	0.2
	rs1806153	11	31850105	1.2×10^{-9}	T	G	1.04	0.0059	0.22
	rs4074723	12	23947737	3.1×10^{-8}	A	C	0.97	0.0049	0.41
	rs4143229	13	44327799	2.5×10^{-8}	A	C	0.95	0.0091	0.92
	rs12552	13	53625781	6.1×10^{-19}	A	G	1.04	0.0048	0.44
	rs4904738	14	42179732	2.6×10^{-9}	T	C	0.97	0.0049	0.57
	rs915057	14	64686207	7.6×10^{-10}	A	G	0.97	0.0049	0.42
	rs10149470	14	104017953	3.1×10^{-9}	A	G	0.97	0.0049	0.49
	rs8025231	15	37648402	2.4×10^{-12}	A	C	0.97	0.0048	0.57

	rs8063603	16	6310645	6.9×10^{-9}	A	G	0.97	0.0053	0.65
	rs7198928	16	7666402	1.0×10^{-8}	T	C	1.03	0.005	0.62
	rs7200826	16	13066833	2.4×10^{-8}	T	C	1.03	0.0055	0.25
	rs11643192	16	72214276	3.4×10^{-8}	A	C	1.03	0.0049	0.41
	rs17727765	17	27576962	8.5×10^{-9}	T	C	0.95	0.0088	0.92
	rs62099069	18	36883737	1.3×10^{-8}	A	T	0.97	0.0049	0.42
	rs11663393	18	50614732	1.6×10^{-8}	A	G	1.03	0.0049	0.45
	rs1833288	18	52517906	2.6×10^{-8}	A	G	1.03	0.0054	0.72
	rs12958048	18	53101598	3.6×10^{-11}	A	G	1.03	0.0051	0.33
	rs5758265	22	41617897	7.6×10^{-9}	A	G	1.03	0.0054	0.28
Anxiety	rs79928194	2	233649290	1.3×10^{-6}	T	C	0.862	0.0515	0.905
	rs342422	5	83470986	1.3×10^{-6}	A	G	0.92	0.036	0.53
	rs2451828	5	7748796	7.4×10^{-7}	T	C	1.34	0.156	0.019
	rs6462203	7	3676002	1.1×10^{-7}	A	C	0.901	0.0345	0.265
	rs16916239	8	87643741	9.0×10^{-7}	A	G	0.903	0.037	0.783
	rs113209956	9	2511193	6.4×10^{-8}	T	C	0.828	0.057	0.085
	rs1458103	11	81047274	6.2×10^{-8}	A	C	0.898	0.0345	0.741
	rs11855560	15	41024303	7.0×10^{-7}	T	C	1.089	0.0365	0.469
	rs6030245	20	41070559	5.1×10^{-7}	T	C	1.12	0.049	0.795
irritability	rs10905638	1	98499843	7.0×10^{-10}	C	G	1.01	0.0013	0.44
	rs11948261	1	2413634	1.2×10^{-10}	A	T	1.01	0.001	0.21
	rs11992186	2	44992550	1.1×10^{-21}	C	G	1.01	0.0013	0.49
	rs13061614	2	45157336	1.2×10^{-8}	C	T	1.01	0.001	0.41
	rs13157212	2	205116051	6.4×10^{-10}	C	A	0.99	0.0011	0.13
	rs1384015	2	122663627	7.2×10^{-9}	T	C	0.99	0.0011	0.74
	rs16884419	3	35694457	7.4×10^{-10}	A	G	1.01	0.001	0.24
	rs1782170	4	147560199	2.5×10^{-8}	C	A	1.01	0.0019	0.72
	rs1782813	5	107753691	1.7×10^{-8}	C	T	1.01	0.0012	0.83
	rs343936	5	46055494	9.0×10^{-11}	C	T	0.99	0.0014	0.17
	rs35733768	7	139835245	5.6×10^{-9}	A	G	0.99	0.0019	0.07
	rs4903249	8	8524474	1.4×10^{-8}	T	C	1.01	0.001	0.49
	rs4953150	8	89579649	1.4×10^{-8}	T	C	1.01	0.0011	0.34
	rs62491417	9	135301389	3.8×10^{-9}	A	C	0.99	0.0011	0.07
	rs6507216	10	9951081	2.4×10^{-8}	T	G	1.01	0.0010	0.34
	rs6711058	11	13267489	3.2×10^{-8}	A	G	1.01	0.0011	0.74
	rs6718682	12	109862335	9.7×10^{-11}	T	C	0.99	0.001	0.28
	rs7970482	14	41674903	5.6×10^{-10}	A	G	1.01	0.0011	0.31
	rs8091413	14	75083881	6.4×10^{-10}	G	C	0.99	0.001	0.74

rs942817	18	35195719	2.4×10^{-8}	C	G	1.01	0.001	0.61
rs999483	18	63549860	6.4×10^{-14}	G	T	1.01	0.0011	0.25

Table S3-S8 Subgroup analyses

Table S3 Results of logistic regression analysis for depression in the middle-aged subgroup (45-59 years)(n=146579)

Variable	Model1		Model2		Model3		Model4	
	OR	S.E.	OR	S.E.	OR	S.E.	OR	S.E.
sociability#education								
1#1							1.084	0.125
1#2							0.997	0.080
1#3							1.137	0.093
sociability#householdincome								
1#1					0.968	0.071		
1#2					1.059	0.075		
1#3					1.157	0.089		
1#4					1.268	0.174		
Sociability#deprivation			0.964***	0.050				
Sociability score(Low)								
High	0.330***	0.009	0.336***	0.012	0.316***	0.016	0.313***	0.022
Deprivation index(Low)								
High	1.291***	0.030	1.302***	0.034	1.291***	0.030	1.291***	0.030
Household income(<18,000)								
18,000-30,999	0.531***	0.017	0.531***	0.017	0.535***	0.019	0.531***	0.017
31,000-51,999	0.406***	0.013	0.406***	0.013	0.400***	0.014	0.406***	0.013
52,000-10,000	0.306***	0.011	0.306***	0.011	0.296***	0.012	0.307***	0.011
>10,000	0.215***	0.014	0.215***	0.014	0.202***	0.015	0.215***	0.014
Education attainment(None of the above)								
Work-related practical qualifications	0.879*	0.044	0.879***	0.044	0.878*	0.044	0.862*	0.050
Secondary	0.780***	0.027	0.780***	0.027	0.780***	0.027	0.781***	0.031
Higher education	0.743***	0.028	0.743***	0.028	0.743***	0.028	0.720***	0.030

Table S4 Results of logistic regression analysis for anxiety in the middle-aged subgroup (45-59 years)(n=146579)

Variable	Model1		Model2		Model3		Model4	
	OR	S.E.	OR	S.E.	OR	S.E.	OR	S.E.
sociability#education								
1#1							0.895	0.083
1#2							1.035	0.065
1#3							1.053	0.067
sociability#householdincome								
1#1					1.087	0.064		
1#2					1.176	0.065		
1#3					1.182	0.069		
1#4					1.248	0.121		
Sociability#deprivation			0.907*	0.035				
Sociability score(Low)								
High	0.398***	0.008	0.416***	0.011	0.355***	0.015	0.388***	0.022
Deprivation index(Low)								
High	1.205***	0.022	1.239***	0.026	1.205***	0.022	1.205***	0.022
Household income(<18,000)								
18,000-30,999	0.628***	0.017	0.628***	0.017	0.613***	0.019	0.628***	0.017
31,000-51,999	0.529***	0.014	0.529***	0.014	0.505***	0.015	0.529***	0.014
52,000-10,000	0.439***	0.012	0.439***	0.012	0.418***	0.014	0.439***	0.012
>10,000	0.318***	0.015	0.318***	0.015	0.298***	0.016	0.318***	0.015
Education attainment(None of the above)								
Work-related practical qualifications	0.907*	0.038	0.907*	0.038	0.906*	0.038	0.937	0.047
Secondary	0.832***	0.024	0.832***	0.024	0.832***	0.024	0.823***	0.028
Higher education	0.794***	0.025	0.794***	0.025	0.794***	0.025	0.782***	0.028

Table S5 Results of logistic regression analysis for irritability in the middle-aged subgroup (45-59 years)(n=146579)

Variable	Model1			Model2			Model3			Model4		
	Median (OR)	significant proportion	positive effect proportion	Median (OR)	significant proportion	positive effect proportion	Median (OR)	significant proportion	positive effect proportion	Median (OR)	significant proportion	positive effect proportion
sociability#education												
1#1										0.871	0	66%
1#2										2.286	0	72%
1#3										2.175	0	73%
sociability#householdincome												
1#1							0.707	0	58%			
1#2							2.285	0	78%			
1#3							0.266	0	80%			
1#4							8.041	0	85%			
Sociability#deprivation				1.573	0	66%						
Sociability score(Low)												
High	0.787	0	82%	0.729	0	86%	0.950	0	54%	0.377	0	78%
Deprivation index(Low)												
High	0.446	4%	100%	0.437	2%	92%	0.441	2%	100%	0.489	4%	100%
Household income(<18,000)												
18,000-30,999	1.482	0	64%	1.411	0	66%	1.555	0	68%	1.557	0	68%
31,000-51,999	1.337	0	68%	1.313	0	70%	0.860	0	60%	1.341	0	72%
52,000-10,000	1.003	0	50%	0.937	0	52%	1.392	0	66%	0.983	0	50%
>10,000	2.340	0	82%	2.516	0	82%	0.808	0	56%	2.749	2%	84%
Education attainment(None of the above)												
Work-related practical qualifications	1.641	0	68%	1.675	0	66%	2.462	0	74%	2.223	0	78%
Secondary	1.157	0	56%	1.165	0	60%	1.246	0	62%	0.648	0	62%
Higher education	1.054	0	52%	1.023	0	50%	1.175	0	56%	0.648	0	66%

Table S6 Results of logistic regression analysis for depression in the middle-aged subgroup (⩾60 years)(n=121564)

Variable	Model1		Model2		Model3		Model4	
			OR	S.E.	OR	S.E.	OR	S.E.
sociability#education								
1#1							1.075	0.134
1#2							1.047	0.085
1#3							1.130	0.092
sociability#householdincome								
1#1					1.278***	0.096		
1#2					1.375***	0.128		
1#3					1.363**	0.178		
1#4					1.535	0.437		
Sociability#deprivation			0.811***	0.052				
Sociability score(Low)								
High	0.354***	0.011	0.389***	0.017	0.304	0.015	0.335***	0.019
Deprivation index(Low)								
High	1.426***	0.044	1.526***	0.057	1.427	0.044	1.426***	0.044
Household income(<18,000)								
18,000-30,999	0.646***	0.024	0.646***	0.024	0.597***	0.027	0.646***	0.024
31,000-51,999	0.529***	0.025	0.529***	0.025	0.477***	0.027	0.529***	0.025
52,000-10,000	0.453***	0.029	0.453***	0.029	0.411***	0.032	0.453***	0.029
>10,000	0.324***	0.046	0.324***	0.046	0.280***	0.050	0.324***	0.046
Education attainment(None of the above)								
Work-related practical qualifications	0.895	0.053	0.896	0.053	0.897	0.054	0.875	0.064
Secondary	0.773***	0.030	0.773***	0.030	0.773***	0.030	0.762***	0.036
Higher education	0.764	0.032	0.764***	0.032	0.764***	0.032	0.734***	0.036

Table S7 Results of logistic regression analysis for anxiety in the middle-aged subgroup (⩾60 years)(n=121564)

Variable	Model1		Model2		Model3		Model4	
	OR	S.E.	OR	S.E.	OR	S.E.	OR	S.E.
sociability#education								
1#1							1.126	0.109
1#2							1.000	0.062
1#3							1.056	0.066
sociability#householdincome								
1#1					1.067	0.062		
1#2					1.210**	0.084		
1#3					0.970	0.093		
1#4					1.024	0.215		
Sociability#deprivation			0.941	0.047				
Sociability score(Low)								
High	0.390***	0.010	0.400***	0.013	0.371***	0.014	0.380***	0.017
Deprivation index(Low)								
High	1.262***	0.031	1.289***	0.039	1.262***	0.031	1.262***	0.031
Household income(<18,000)								
18,000-30,999	0.696***	0.020	0.696***	0.020	0.680***	0.024	0.696***	0.020
31,000-51,999	0.597***	0.021	0.597***	0.021	0.557***	0.025	0.597***	0.021
52,000-10,000	0.555***	0.027	0.555***	0.027	0.560***	0.032	0.555***	0.027
>10,000	0.397***	0.040	0.397***	0.040	0.394***	0.049	0.397***	0.040
Education attainment(None of the above)								
Work-related practical qualifications	0.905*	0.043	0.905*	0.043	0.905*	0.043	0.865*	0.052
Secondary	0.849***	0.026	0.849***	0.026	0.849***	0.026	0.849***	0.032
Higher education	0.826***	0.027	0.826***	0.027	0.825***	0.027	0.810***	0.033

Table S8 Results of logistic regression analysis for irritability in the middle-aged subgroup (⩾60 years)(n=121564)

Variable	Model1			Model2			Model3			Model4		
	Median (OR)	significant proportion	positive effect proportion	Median (OR)	significant proportion	positive effect proportion	Median (OR)	significant proportion	positive effect proportion	Median (OR)	significant proportion	positive effect proportion
sociability#education												
1#1										0.033	12%	100%
1#2										0.0627	60%	100%
1#3										0.230	2%	96%
sociability#householdincome												
1#1							0.360	0	98%			
1#2							0.362	2%	88%			
1#3							0.924	0	54%			
1#4							0.633	0	62%			
Sociability#deprivation				1.327	0	72%						
Sociability score(Low)												
High	0.412	34%	100%	0.323	34%	100%	0.687	0	84%	1.901	0	78%
Deprivation index(Low)												
High	1.138	0	70%	0.946	0	54%	1.147	0	66%	1.204	0	62%
Household income(<18,000)												
18,000-30,999	1.170	0	60%	1.140	0	62%	1.882	0	82%	1.289	0	70%
31,000-51,999	0.820	0	68%	0.858	0	68%	1.230	0	78%	0.913	0	54%
52,000-10,000	1.311	0	62%	1.325	0	66%	1.600	0	68%	1.278	0	58%
>10,000	1.602	0	82%	1.500	0	80%	2.485	0	92%	1.677	0	68%
Education attainment(None of the above)												
Work-related practical qualifications	0.633	0	82%	0.618	0	86%	0.705	0	82%	2.601	2%	96%
Secondary	0.778	2%	78%	0.847	0	76%	0.868	0	78%	2.947	6%	94%
Higher education	0.243	36%	100%	0.254	36%	100%	0.272	32%	100%	0.620	0	82%
